# Supplementary material for: General Markers of Conscious Visual Perception and Their Timing
Source: Front Hum Neurosci. 2016 Feb 4;10:23. doi: 10.3389/fnhum.2016.00023 (PMC4740392; doi:10.3389/fnhum.2016.00023)
Supplement: Supplementary file 1 [file Presentation_1.PDF]

S1 Pre-experiment

Not all of the 70 stimuli have the same threshold contrast. An earlier pilot experiment indicated that there are 5 groups of stimuli with roughly similar threshold contrasts within each group: 1) text; 2) solid graphical figures; 3) line-drawings of graphical figures; 4) solid forms of all other figures and 5) line-drawings of all other figures. The appropriate contrasts for these 5 groups of stimuli were determined with the help of a short pre-experiment prior to the main experiment. The pre-experiment was very similar to the main experiment (see "2.3 Task and design"), except that a separate set of stimuli including all the 10 stimulus types (see "2.2 Stimuli") was used. Each stimulus (19 in total) was presented twice on 4 adjacent contrast levels. The specific contrast levels were different for each of the 5 contrast groups. They were typical threshold contrasts (i.e. lead to 50% seen responses) for these groups as indicated by an earlier pilot experiment. Subjects had to report whether they perceived a stimulus on each trial. Based on the detection rates of the pre-experiment, individual threshold contrasts for each of the 5 groups of stimuli were estimated by the experimenter. Occasionally, some of the contrasts had to be readjusted after the first block of the main experiment if detection rate was lower than 25% or higher than 75% for a particular group of stimuli.

S2 Physical characteristics of stimuli

Stimuli were presented on a light gray background with a luminance of 51.6 cd/m^2. The space-average luminance of the stimuli per contrast group (see S1) was: group 1 = 49 cd/m^2, group 2 = 46.6 cd/m^2, group 3 = 49.2 cd/m^2, group 4 = 48.2 cd/m^2, group 5 = 48.9 cd/m^2. The size of the stimuli was approximately 2.5 degrees of visual angle. Prior to the stimulus a fixation cross was presented. The size of the fixation cross was 0.35 degrees of visual angle and its luminance was 11.4 cd/m^2. The response screen contained the question “Did you see something?” in the Estonian language. The contrast of the text was also low (luminance of 24 cd/m^2), in order not to disturb the adaption of the eyes for very low contrast stimuli.

Mean RMS contrast for each contrast group (see S1) separately are: group 1 = 0.014, group 2 = 0.017, group 3 = 0.013, group 4 = 0.016, group 5 = 0.011. Note that RMS contrast is calculated for the original 290x290 pixel images and averaged over the individually set threshold contrasts for each subject. Because pixel counts of images were kept very similar within each contrast group and thus SD are very small, only mean RMS contrast values are reported.

Figure A depicts the spatial frequency composition of the stimuli per category (on log-log scale). We compared each stimulus category against the average power spectrum over all stimuli. Images of solid forms (lines “2”, ”5”, ”8” and “10” in the upper panel) were strongly dominated by spatial frequencies below ca. 2 Hz per degree of visual angle – below 2.8 deg for solid graphical figures (“2”), below 1.5 deg for solid forms of man-made objects (“5”), below 2.2 deg for solid forms of animated and inanimate nature (“8” and “10”, respectively). Images of short words (line “3” in the upper panel) were characterized by above average power for spatial frequencies between 1.2-3.4 Hz per degree of visual angle. The spatial frequency composition was also quite similar for all line drawings except line-drawings of graphical figures (see lower panel of figure A). They exhibited higher power in spatial frequencies above ca. 3 Hz per degree of visual angle – above 2.7 deg for line-drawings of man-made objects (“4”), above 2.9 deg for line-drawings of faces (“6”) , above 3.4 deg for line-drawings of animated and inanimate nature (“6” and “9”, respectively). Line-drawings of graphical figures (“1”), on the other hand, were characterized by above average power for spatial frequencies between 1.7 – 8.8 and 16.5 – 26.5 3 Hz per degree of visual angle only.

From the above described characteristics of the employed heterogeneous stimulus set it is quite obvious (and nothing less than expected) that there are systematic differences in the visual features of different stimulus categories. Despite these differences between stimuli and stimulus categories, however, one can be certain that any variability in the EEG results does not stem from them. This is because we use an additional trial matching procedure which makes sure that all matched subsets of the data are

objectively identical with regard to stimulus content and stimulus features (see “2.5.1 Trial matching procedure”).

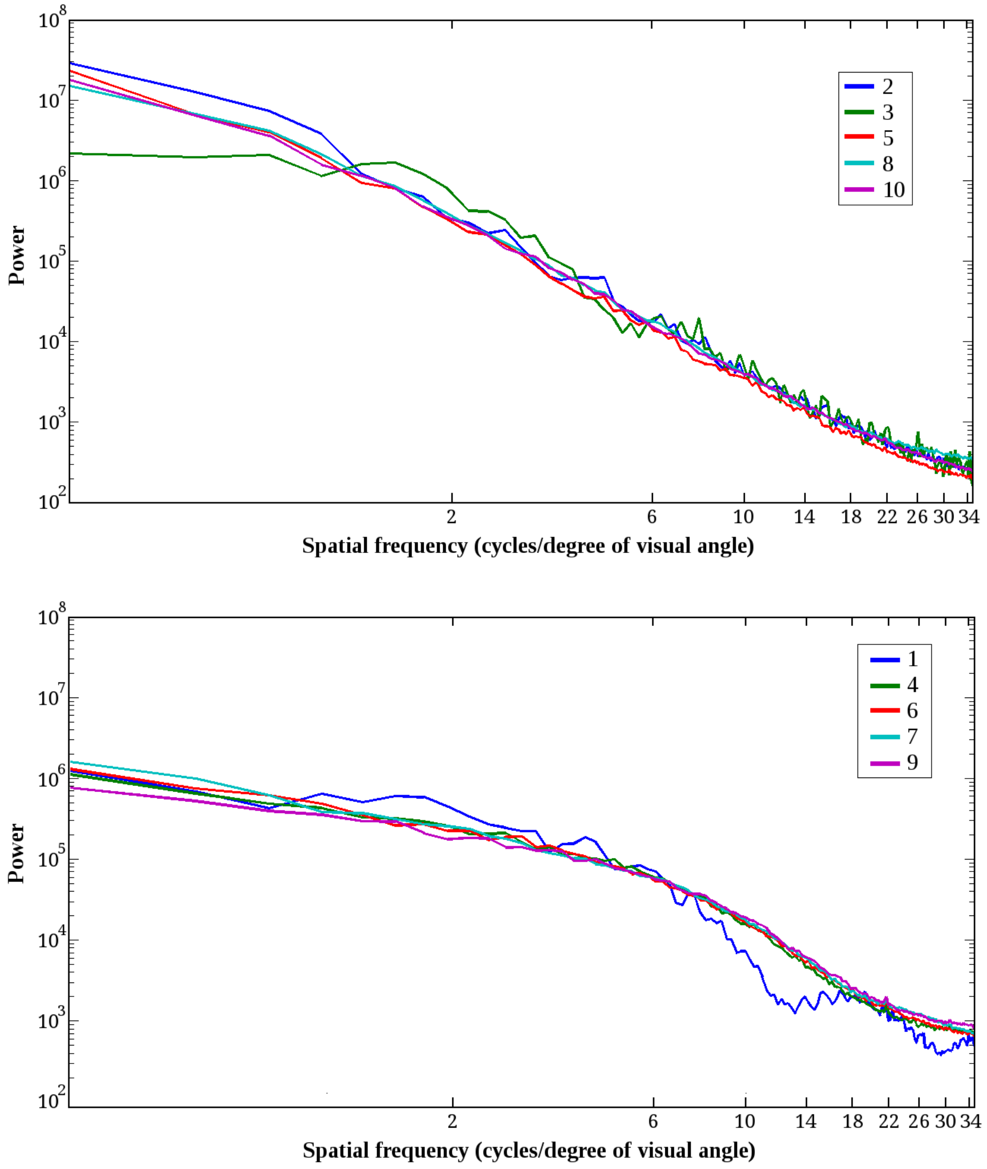

Figure A. Spatial frequencies per stimulus category. The frequency spectra are plotted on a log-log scale. The upper panel depicts information for images of solid forms and short words. The lower panel depicts information for all line drawings. Line numbers refer to the image category numbers as listed in “2.2 Stimuli”.

S3 Neighboring electrodes and cluster formation

For each of the 60 electrodes its neighboring i.e. surrounding electrodes are defined within a fixed radius. Note, however, that the radius varies depending on cap size (S =3.5 cm, M = 3.7 cm, L = 4 cm). As a consequence, each electrode has maximally 4 nearest neighbors, one in each cardinal direction. But lateral electrodes have less neighbors of course. For example, 'Cz' has neighbors 'FCz', 'C1', 'C2' and 'CPz', but 'Iz' only has 'Oz' as a neighbor.

This neighborhood structure is used by the cluster permutation test algorithm to group together data points that exhibit significant differences between conditions IF these data points are neighbors in time AND in space (i.e. they occur on neighboring electrodes). Figure B shows one such cluster. It is the P300 component that exhibited significant differences between the seen and the unseen condition. Note that, although not all rows are next to each other in the two-dimensional electrode-by-time plot, the corresponding electrodes are actually neighbors on the EEG cap. They were therefore clustered together.

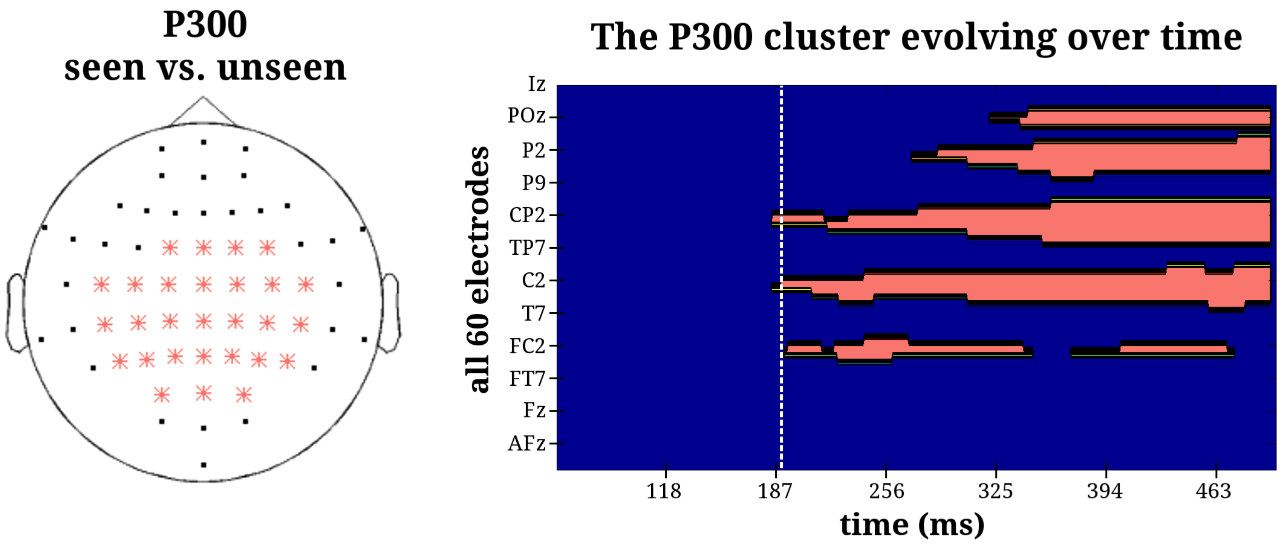

Figure B. Typical result of a cluster permutation test. On the right hand side is the electrode-by-time representation of the significant cluster (i.e. P300). All data points (electrode-time pairs) where the seen and the unseen condition exhibited significant differences are colored pink. Together they form the P300 cluster. The onset latency of this cluster is marked with the vertical dashed white line at 190 ms. This is the first time point where at least 4 neighboring electrodes show significant differences between conditions. On the left hand side are all electrodes belonging to the P300 cluster.

S4 Denoising of electrodes

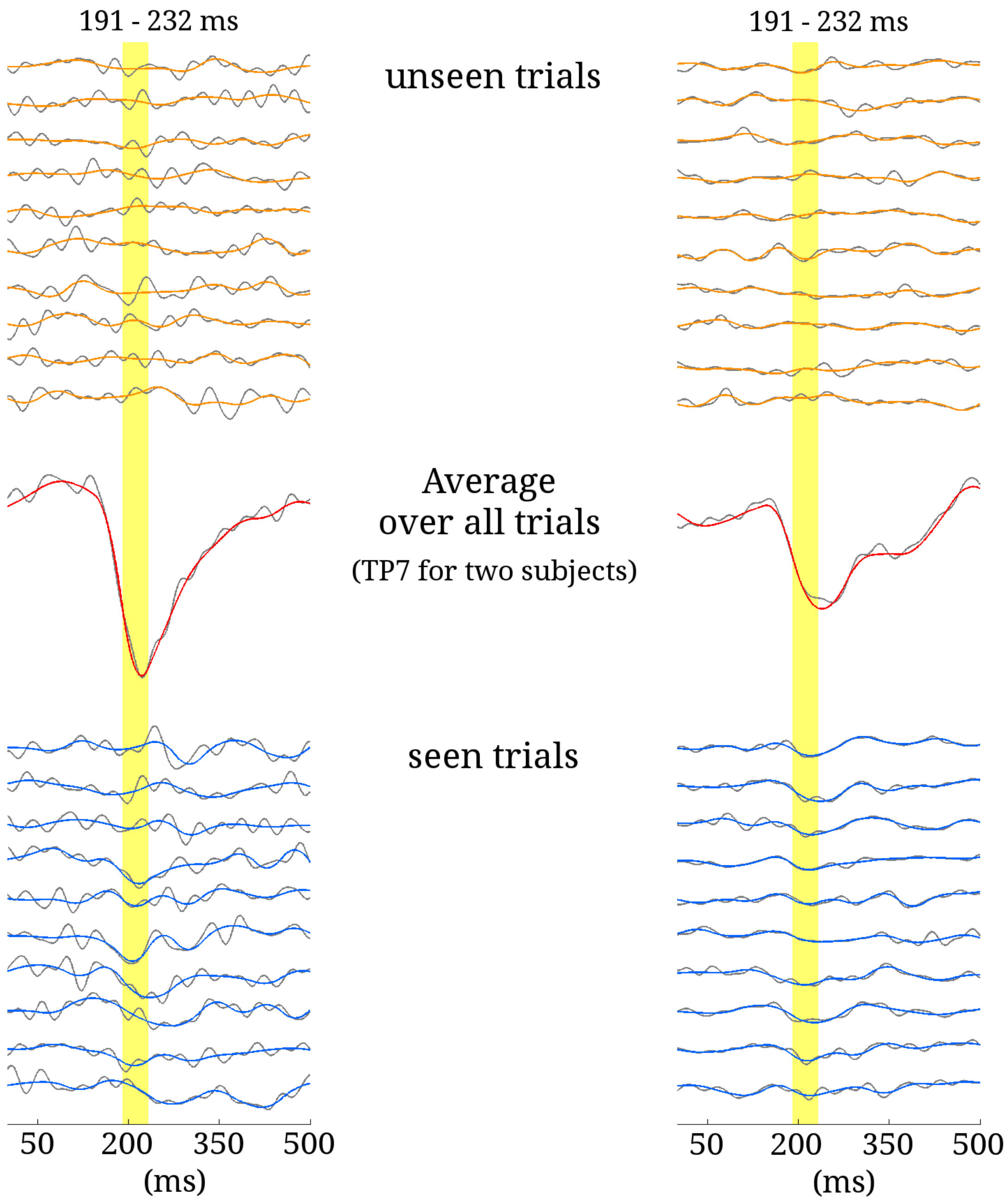

Figure C. Examples of denoised trials. The left- and right-hand side depict data from two different subjects, respectively. The ERPs over all available trials (seen, unseen, catch) are depicted in the middle. This is the signal that the denoising algorithm tries to reconstruct in the single trials. Some exemplary unseen trials are illustrated above the ERPs. Some exemplary seen trials are illustrated below the ERPs. Gray traces depict the original data. Colored traces depict the denoised data.

S5 Correlation tests with ERP parameters

In addition to the 12 correlation tests described in “2.5.4 Correlation tests” four extra correlation test were carried out between averaged ERP parameters and cluster onset latencies. For these tests denoised single trial data was first averaged for each electrode per condition. Then, peak amplitude and peak latency of N200/P300 (depending on the electrode) was noted for the seen and the unseen condition. Finally, these values were averaged over electrodes and over subjects and correlation tests were carried out with the onset latencies of the respective clusters. All the p-values (n = 16) were corrected for multiple comparisons with the Holm-Bonferroni method.

The correlation between mean peak amplitude of the averaged seen trials and the P300 onset times was not significant (r = -0.26, t = -2.63, p = 0.089), but the correlation for mean peak amplitude of the averaged unseen trials was again significant (r = 0.53, t = 6.17, p = 2.5e-07). Similarly, the correlation of mean peak amplitude with N200 onset times for the seen trials was only marginally significant (r = 0.28, t = 2.9, p = 0.049). The same correlation for unseen trials was again significant (r = -0.47, t = -5.14, p = 1.9e-05). Figure D illustrates the results for these correlation tests.

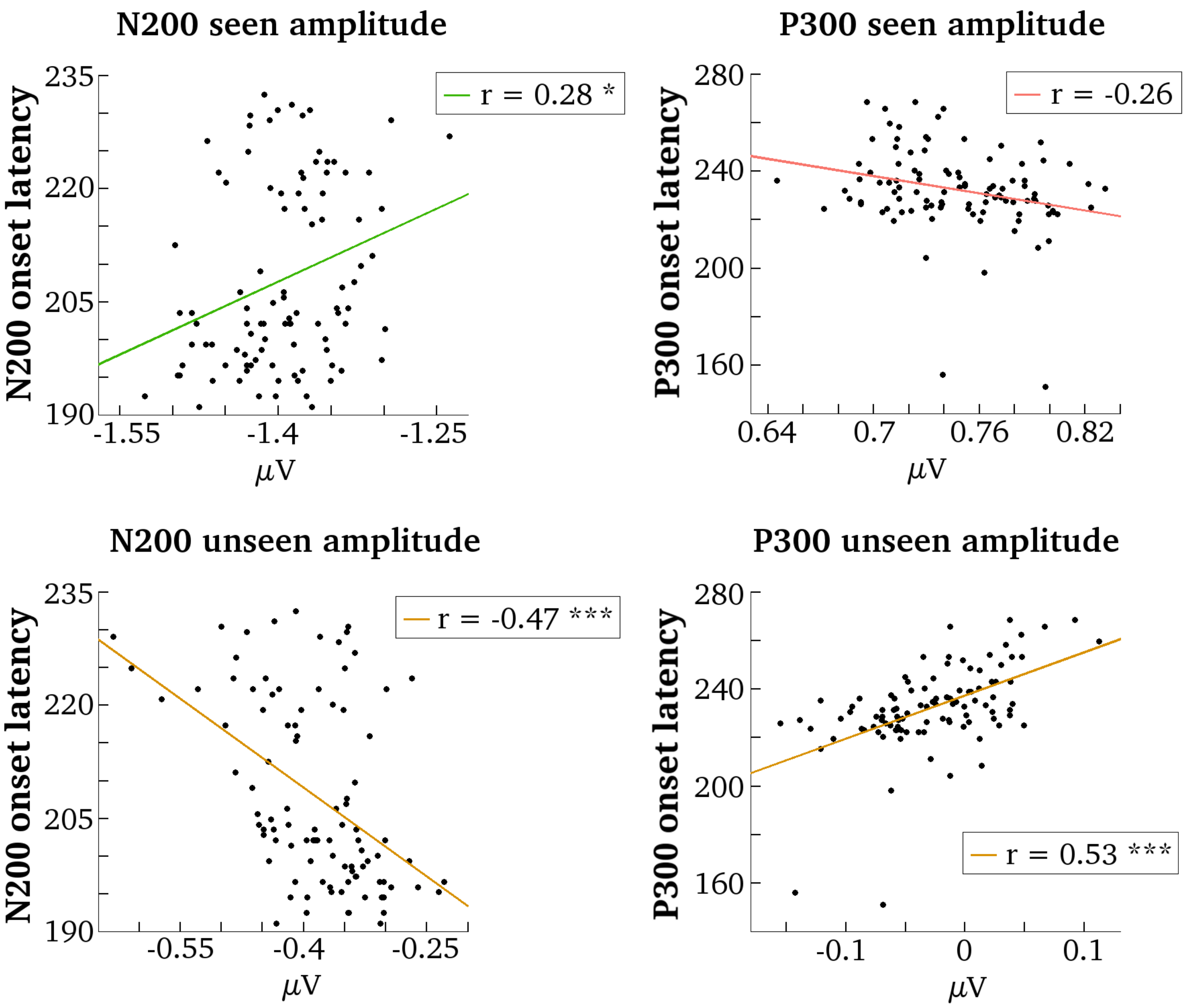

Figure D. Correlations between grand averages of N200 and P300 amplitudes (after averaging the single trial responses per subject) and the respective gmNCC onset times (indicated in ms on the y-axes). Correlation tests are carried out separately for seen and unseen trials. P-values < 0.05 are indicated with *. P-values < 0.001 are indicated with ***.

S6 Detection rates for all stimulus types separately

1. graphical line-drawings ($m = 0.57$, $SD = 0.26$)
2. graphical solid forms ($m = 0.39$, $SD = 0.29$)
3. short words ($m = 0.87$, $SD = 0.19$)
4. line-drawings of man-made objects ($m = 0.5$, $SD = 0.2$)
5. solid forms of man-made objects ($m = 0.47$, $SD = 0.37$)
6. line-drawings of faces ($m = 0.49$, $SD = 0.2$)
7. line-drawings of animated nature ($m = 0.49$, $SD = 0.2$)
8. solid forms of animated nature ($m = 0.48$, $SD = 0.36$)
9. line-drawings of inanimate nature ($m = 0.36$, $SD = 0.2$)
10. solid forms of inanimate nature ($m = 0.47$, $SD = 0.38$)

It is evident that the percentage of successfully perceived stimuli varies considerably between different stimulus types and even between single exemplars within a stimulus type. This, however, is not a problem for our present study. We are interested in the general markers of conscious visual perception. Such markers should not be affected by stimulus content variability. On the contrary, variance between stimuli can only strengthen any conclusions drawn from the results.

Furthermore, an ANOVA with factors stimulus type and conscious perception did not reveal any systematic effects on the proportion of trial numbers (main effect for stimulus type: $F(8,136) < 1.0$; main effect for conscious perception: $F(1,17) < 1.0$; interaction: $F(8,136) = 1.9$, $p = 0.07$). Note that for this ANOVA the stimulus type “short words” was excluded because it is already known that for this type detection rate is much higher than 50% on average.

S7 Late negativity

The late negativity constituted a significant cluster on fronto-temporal electrodes. Like the P300, this negative cluster was significant on all 100 iterations and comprised of 21 electrodes on average (median = 21, SD = 0.4, range = 20 - 22). The mean onset latency of statistical significance for this negative cluster was 307 ms (median = 309, SD = 11 ms, range = 283 - 334 ms). Fig. D contains a histogram of the distribution. Again, this cluster was always significant until the end of the tested time period.

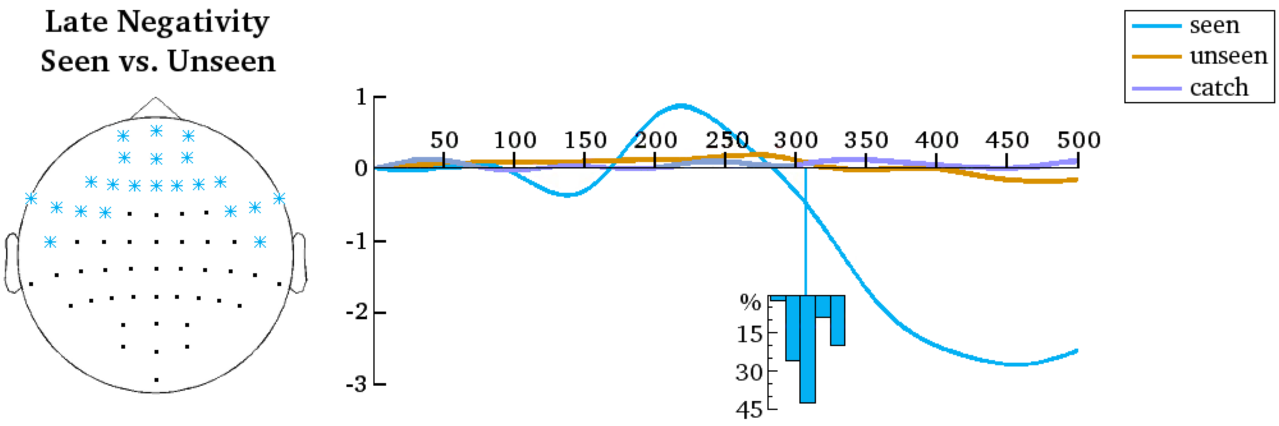

Figure E. Summarized results for the late negativity. ERPs are averaged over the indicated electrodes (left). These are all the electrodes that belonged to the respective cluster for at least 1 of the 100 contrastive analyses between the seen and the unseen condition. The histogram depicts the distribution of cluster onset times over the 100 contrastive analyses. Note that the distribution aligns with the time axis (in ms).

S8 Comparisons between the seen condition and baseline

For comparisons between the seen condition with the baseline of the unseen condition post-stimulus data of the unseen condition was replaced by pre-stimulus data from the same trials. The same electrodes as for the main analysis were used. To remove any potential real components like CNV the baseline data was first detrended. Then all analysis steps were repeated just like before. The data was denoised and all the 100 contrastive analysis were carried out on the same sets of matched trials. Correlation tests with gmNCC onset latencies were conducted for mean peak amplitude, mean peak latency and standard deviation of peak latency from the seen trials and from the unseen trials (i.e. baseline activity) in the new time windows of observed variability in gmNCC onset latencies. The presently reported p-values are also corrected for 16 comparisons to make them comparable to the main results, but the significant changes in correlations with mean peak amplitude from the baseline activity are already visible when only the correlation coefficients are considered.

P300 was again significant on 100% of the iterations and always included all 9 electrodes that were selected for denoising. The new mean onset latency of P300 was 205 ms (median = 206 ms, SD = 8 ms, range = 180 - 219 ms) and P300 again always remained significant until the end of the tested time period. Compared to the results in the main text, variability in P300 onset latencies was now best correlated with the mean peak amplitude of the seen trials (r = -0.3, t = 3.14, p = 0.03). The correlation with mean peak amplitude of the "unseen" trials was not significant any more (r = 0.2, t = 2.03, p = 0.54). The onset times of P300 did not correlate significantly with mean peak latency for the seen nor for the "unseen" trials (r = -0.15, t = -1.47, p = 1.0 and r = 0.13, t = 1.33, p = 1.0, respectively). The correlations with mean latency variance were also not significant (r = 0.13, t = 1.25, p = 1.0 for seen trials; r = -0.08, t = -0.77, p = 1.0 for "unseen" trials). Figure F contains histograms of the respective distributions and correlations for mean peak amplitude.

N200 was also significant on 100% of the iterations and always included all 10 electrodes that were selected for denoising. Thus, compared to the results in the main text N200 is more reliably correlated with the seen condition. The new mean onset latency of N200 was 191 ms (median = 190 ms, SD = 3 ms, range = 179 - 198 ms). The new mean offset latency was 331 ms (median = 330 ms, SD = 3 ms, range = 325 - 337 ms). Thus, the new mean duration of the N200 was 140 ms (median = 139 ms, SD = 4 ms , range = 130 – 154 ms). Importantly, variability in N200 onset latencies was now best correlated with the mean peak amplitude of the seen trials (r = 0.39, t = 4.14, p = 0.001). The correlation with mean peak amplitude of the "unseen" trials was not significant any more (r = -0.26, t = -2.64, p = 0.13). The onset times of N200 did not correlate significantly with mean peak latency for the seen nor for the "unseen" trials (r = -0.13, t = -1.25, p = 1.0 and r = -0.15, t = -1.44, p = 0.32, respectively). The

correlations with mean latency variance were also not significant (r = 0.13, t = 1.29, p = 1.0 for seen trials; r = 0.01, t = 0.1, p = 1.0 for “unseen” trials). Figure F contains histograms of the respective distributions and correlations for mean peak amplitude.

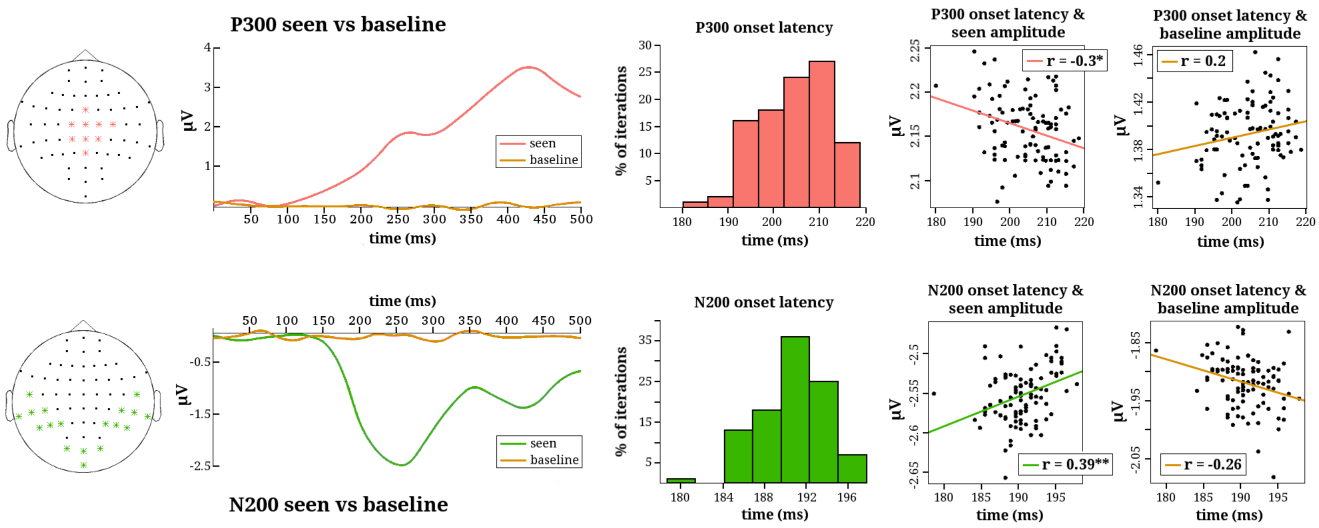

Figure F. Results for the seen condition vs. baseline. Denoised data is averaged over the indicated electrodes (left) and plotted over time for the seen condition and for baseline activity of the “unseen” condition (second from left). Histograms depict the distributions of gmNCC onset times over the 100 different contrastive analyses (third from left). The right hand side shows correlations between averages of single trial N200/P300 mean peak amplitudes and N200/P300 onset times (indicated in ms on the x-axes). Correlation tests are carried out separately for seen trials and baseline activity of the “unseen” trials. P-values < 0.05 are indicated with *. P-values < 0.01 are indicated with **.

S9 GmNCC for different stimulus classes and detection rates

In order to investigate if certain stimulus characteristics have a reliable effect on N200/P300 parameters during the time period of their onset dependent samples t-tests comparing different groups of stimuli were carried out. First, only seen trials were considered in these analyses, because N200 and P300 seem to be uniquely associated with the seen condition. Second, all seen trials with extreme peak amplitude values during the time period of cluster onsets were removed from the data. Thus, all seen trials with peak amplitude values above or below two standard deviations from the grand average of peak amplitude were removed for the present analyses. Third, all seen text stimuli were removed for the present analyses, because stimuli of this type were detected more often than stimuli of other types. It is preferable to avoid this systematic unbalance between stimulus types.

One set of dependent samples t-tests was carried out to compare N200/P300 parameters for line-drawings and solid form images. The first group (i.e. line-drawings) consisted of all trials where line-drawings of graphical figures, man-made objects, animated nature, or inanimate nature were presented. The second group (i.e. solid forms) included all trials where solid form images of graphical figures, man-made objects, animated nature, or inanimate nature were presented. Thus, in addition to text stimuli, face stimuli were also excluded from this set of t-tests, because there was no solid form equivalent for face stimuli. On average, the were 114 trials available for line-drawings (median = 110, SD = 42.2, range = 45 - 228) and 101 trials for solid forms (median = 85, SD = 43.8, range = 38 - 164).

Another set of dependent samples t-tests was carried out to compare N200/P300 parameters for stimuli with higher and lower detection rates. The first group (i.e. high detection rate) consisted of those trials where a stimulus with > 50% detection rate was presented (each stimulus was presented 10 times and thus has a detection rate). The second group (i.e. low detection rate) consisted of those trials where a stimulus with <= 50% detection rate was presented. On average, the were 162 trials available for the high detection rate condition (median = 150, SD = 89.4, range = 40 - 367) and 83 trials for the low detection rate condition (median = 90, SD = 28.2, range = 31 – 131).

For both sets of t-tests mean peak amplitude, mean peak latency and the standard deviation of peak latency (for both the N200 and the P300) were compared between groups. Note that as for the correlation tests in "3.4 gmNCC onset variability explained by single trial parameters", these parameters were extracted from denoised single trials and averaged over the same representative electrodes of the respective clusters (see "2.5.4 Correlation tests" for more information). The results are presented in Table A below.

	Peak Amplitude	Peak Latency	Peak Latency SD
	**T-tests for N200**		
Stim. class	t = -2.0, p = 0.44, ges = 0.06	t = -0.44, p = 1.0, ges = 0.003	T = -1.98, p = 0.44, ges = 0.03
Det. rate	t = 3.49, p = 0.03, ges = 0.11	T = -1.15, p = 1.0, ges = 0.02	T = 0.36, p = 1.0, ges = 0.002
	**T-tests for P300**		
Stim. class	T = 2.87, p = 0.1, ges = 0.04	T = 0.08, p = 1.0, ges = 0.0001	T = -0.69, p = 1.0, ges = 0.009
Det. rate	T = -3.38, p = 0.04, ges = 0.05	T = -3.53, p = 0.03, ges = 0.16	T = 2.5, p = 0.18, ges = 0.06

Table A. All t-tests have 17 degrees of freedom. All p-values are Holm-Bonferroni corrected for 12 tests in total. P-values < 0.05 are marked yellow. For effect size estimates we report generalized eta squared (ges).
